# Favism: Clinical Features at Different Ages

**DOI:** 10.3390/nu15020343

**Published:** 2023-01-10

**Authors:** Alice Beretta, Matteo Manuelli, Hellas Cena

**Affiliations:** 1 Laboratory of Dietetics and Clinical Nutrition, Department of Public Health, Experimental and Forensic Medicine, University of Pavia, Via Bassi 21, 27100 Pavia, Italy; 2 Clinical Nutrition and Dietetics Service, Unit of Internal Medicine and Endocrinology, Istituti Clinici Scientifici Maugeri IRCCS, 27100 Pavia, Italy

**Keywords:** favism, G6PD deficiency, nutrition, hemolysis

## Abstract

Favism is a hemolytic disease due to the ingestion of fava beans in individuals with glucose-6-phosphate dehydrogenase (G6PD) deficiency. There is wide inter- and intra-individual variability in the development of hemolytic crisis, and several factors influence it: quantity, quality, ripeness of fava beans, and age of onset. In this narrative review of case reports and case series, we reported the predisposing factors and clinical features for four different age groups classified as follows: pregnant women and infants (i.e., exclusively breastfed children); children, from weaned to 11 years; preadolescents and adolescents, from 11 to 18 years; and adults (18 years and older). Some symptoms developed only in specific age groups: death in infants; visual impairment in children; systolic murmur in infants, children, and adolescents; and renal failure in adults. In youngest children or pregnant women the severity is the highest. Some other symptoms were present in all: jaundice, increased bilirubin, splenomegaly, hepatomegaly, discolored urine, tachycardia, pallor, abdominal pain, malaise, vomit, nausea, and dizziness. Laboratory findings are characterized by anemia, reticulocytosis, elevated bilirubin level, and sometimes urinary urobilinogen and methemoglobinemia. In most cases the symptomatology is self-limited and does not release sequelae, but hospitalization and transfusion are often required.

## 1. Introduction

Favism is a hemolytic disease due to the ingestion of fava beans in subjects with glucose-6-phosphate dehydrogenase (G6PD) deficiency. This is a common erythrocyte enzyme deficiency with sex-linked inheritance caused by the presence of a mutation in the constitutive Gd gene. G6PD participates in pentose phosphate metabolism, and it is responsible for maintaining an adequate reducing potential in the erythrocyte. Enzymatic variants, resulting from mutations in the polymorphic gene, have different levels of activity which result in different manifestations of the defect at the clinical level. Based on biochemical and clinical characteristics, the WHO [1] has classified G6PD variants into four classes as shown in Table 1.

At steady state, most of the deficit carriers do not show clinical manifestations; the enzymatic defect corresponds to a mild hemolytic state that, in most cases, is perfectly compensated [2]. When an exogenous trigger induces increased oxidative stress in red blood cells, a hemolytic crisis occurs. In the case of favism, the triggers are two components present in fava beans: vicine and convicine. In the intestine, the two beta-glucosides are converted into their respective aglycones, divicine and isouramil. Their action is to increase the production of free radicals that eventually lead to the oxidation of glutathione. The deficiency of G6PD results in a deficiency of NADPH, whose major task is to reduce the oxidized glutathione, especially in red blood cells, which is a strong oxidizing agent and as such can lead to the onset of a hemolytic crisis. The presence of these glucosides is the discriminating factor between broad beans and other legumes; in fact, other legumes do not present the triggering agents [3]. The quantity of vicine and convicine determine the onset or not of the hemolytic crisis and, in the case of crisis, of its severity also [4].

Hemolytic crises are erratic, so not only are there a wide range of interindividual severities of the reaction, but only 25% of deficient adults manifest a hemolytic crisis following fava bean ingestion; even in the same person there is wide variability in the exacerbation of symptomatology [5]. As reported by Luzzatto and Poggi [6], several factors influence whether or not a hemolytic crisis occurs: the amount of fava beans ingested, especially in relation to mass; the quality of the fava beans (i.e., raw, boiled, or canned); and the degree of ripeness, which corresponds to the amount of glucosides present in the legume. Finally, age of onset can influence the clinical presentation. Hereafter, we review studies reporting favism with different ages of onset (see also Table 2).

## 2. Materials and Methods

G6PD deficiency is the most prevalent enzymopathy in the world [2]. Globally, it is the leading cause of neonatal jaundice and acute hemolytic crisis at any age, due to dietary intake of fava beans. The WHO working group stressed the importance of education and information as key tools for prevention of complications of G6PD deficiency [46]. As there are few reviews that unify the knowledge produced to date on the topic of favism, we summarized the literature to answer our research question on the influence of inter- and intra-individual variability in the development of acute hemolytic anemia and several factors related to the quantity, quality, and maturity of fava beans ingested, as well as age of onset, to improve awareness on the topic. The authors A.B., M.M, and H.C. independently reviewed all relevant literature produced from the earliest reports of cases of favism to the present time (i.e., 1945–2021) in the form of case reports and case series. Original papers, meta-analyses, clinical trials, and reviews were excluded. We imposed restrictions for language (English, Italian, Spanish) and species (humans). We carried out a thorough search for relevant papers on three databases: PubMed/MEDLINE, Scopus, and EMBASE. The following keywords (alone and/or in combination) were used for the research: favism, G6PD deficiency, acute hemolysis anemia, hemolysis, nutrition, age, fava beans, type, quantity, ripeness. Results are reported in the form of a narrative review.

## 3. Results

### 3.1. Pregnant Women and Nursed Infants

Only a single case of a pregnant woman diagnosed with favism, whose fava bean intake did not affect her pregnancy, was found in 1962. She was a 35-year-old woman in the sixth month of pregnancy hospitalized for jaundice, caused by a generous portion (two pounds) of fresh fava beans consumed over a day and a half. Physical examination revealed jaundice, malaise, hepatomegaly, splenomegaly, and a systolic murmur; the laboratory findings are shown in Table 2. In this case, no therapy was administered, but the woman was forced to rest, and she was discharged after 16 days. Four months after the hemolytic episode, the patient gave birth to a male who did not show any significant data. The activity of G6PD in the mother’s erythrocytes was 3.7 units (normal 5 to 7 units), while in the newborn it was 8.9 units, which is normal in children in the first few weeks of life. The authors, Cahill KM and Lay AB [7], investigated the level of G6PD activity in the whole family, finding a deficiency only in the mother of the patient.

In contrast to the case above, Mentzer WC and Collier E [8], in 1975, reported the case of a woman with a history of fried fava bean intake during pregnancy, which played an important role in the exacerbation of intrauterine hemolysis. This led to the death of the infant two hours after birth, having never established spontaneous respirations. Family history included a sister who was born with jaundice, a male maternal cousin treated with phototherapy for neonatal jaundice, a maternal aunt who was anemic and presented jaundice for one month at the age of 10, and finally the maternal grandmother who was anemic. In this case, G6PD enzyme activity was investigated in the mother, who was found to be heterozygous for G6PD deficiency but with no abnormal migrating band, and in the child, who was also found to be G6PD deficient. The authors concluded that eating fava beans, while being supplemented with ascorbic acid—considered a mild hemolytic agent in subjects with G6PD deficiency—and having a viral in utero infection, may be responsible for the intrauterine hemolysis.

Fortunately, a pregnant woman’s intake of fava beans when the baby has a G6PD deficiency does not always result in a fatal outcome, as shown in the case reported by Corchia C et al. [9] in 1995. A 2-hour-old female infant, born at 37 weeks of gestation by cesarean section, developed acute hemolytic anemia (AHA) after her mother’s ingestion of cooked dried fava beans five days before delivery. The treatment of the child with a whole blood transfusion and phototherapy lead to a complete remission of symptoms, and so she was discharged on the 11th day of hospitalization in good clinical condition. G6PD activity investigated after four months reported a 4.61 U/g Hb (normal concentration, 6.73 +/− 2.10). G6PD activity was investigated in the entire family and reported the following values: 5.9 U/g Hb in the mother, 7.10 U/g Hb in the father, 0 and 5.4 U/g Hb in the mother’s two brothers, respectively, and 4.68 U/g Hb in the maternal grandmother.

Some case reports and case series describe the effects of fava bean ingestion by lactating women whose children are deficient in the G6PD enzyme, and developed varying degrees of severity in their symptoms.

In 1956, Casper J and Shulman J [10] reported the case of a 6-month-old Yemeni male infant who died three days after admission to the pediatric ward for AHA. Jaundice, hepatomegaly, pallor, vomit, and dark urine began two days after his mother’s ingestion of fava beans. On the day of admission, urination ceased completely, causing an increase in blood urea to 276 mg/100 mL. The infant was treated with a transfusion of 200 mL of blood, but the outcome was negative. He was diagnosed with favism, even though the mother had never shown any signs of G6PD deficiency, on the basis of the history of fava bean consumption and laboratory findings. 

Emanuel B and Schoenfeld A [11] reported, in 1961, the case of an Egyptian female breastfed infant whose mother ate fava beans three days before admission. On physical examination, the infant showed malaise, jaundiced skin, pale mucous membranes, hepatomegaly and splenomegaly, and dark urine with increased urobilinogen content (1:220). Since the erythrocyte count was 1,000,000/mm^3^, the child was transfused twice with whole blood resulting in a return to the normal range of clinical parameters and discharge. In this case, broad beans were a food regularly present in the family diet and there was no history of diagnosed favism. The authors had investigated the level of reduced glutathione after incubation of blood with acetylphenylhydrazine, a characteristic erythrocyte abnormality in favism, which was found to be 41 mg per 100 cc of erythrocytes (normal value: 70 mg per 100 cc). This single value was not enough for a diagnosis of enzymatic abnormality, but it was proven to be a homozygous G6PD deficiency six weeks after the initial episode, thanks to a Beutler test.

Finally, in 1971, Taj-Eldin S [12] reported four cases of diagnosed favism in exclusively breast-fed infants who developed AHA. The first one, a four-month-old Arab boy, was admitted four days after his mother ate boiled fava beans. On physical examination, he showed pallor, malaise, jaundice, hepatomegaly, and dark urine. Laboratory findings showed erythrocytes 1,480,000/mm^3^, hemoglobin 3.8 g/dL, reticulocytes 7%, total bilirubin 4.2 mg/dL, indirect bilirubin 3.6 mg/dL, and a negative indirect Coombs test. The child was treated with a whole blood transfusion, followed by a complete recovery. The other three reported cases were all Arab boys, who first showed symptoms 2–3 days after their mothers ate fava beans. On physical examination, jaundice, dark urine, hepatomegaly or splenomegaly, and pallor were noted. The laboratory findings showed, respectively: hemoglobin 4.7, 5.2 and 4.0 g/dL; erythrocytes 2,100,000, 1,900,000 and 1,210,000/mm^3^; reticulocytes 6%, 12% and 10%; total bilirubin 3.8, 4.5 and 5.0 mg/dL. Urobilinogen was found positive in the urine in case 1 and case 3. No data on the treatment of the latter three cases are reported. G6PD deficiency was demonstrated in all cases with the Motulsky dye test, strongly suggesting the diagnosis.

The symptom spectrum for this age category presented systolic murmurs and fatal renal failure, in addition to the classic symptoms. The time between the ingestion of the triggering substances and the onset of symptoms ranges from a minimum of 24 hours to a maximum of 5 days, and more days are needed for hospitalization. Furthermore, the two reported deaths underline the fact that favic attacks are more commonly severe in children due to the high ratio between ingested causative agents and body mass, and in male children whose mothers are not aware that they are healthy carriers of the recessive gene. Even though the patients are infants, there is no mention of whether or not they had experienced other episodes later in life. All the clinical features of favism in this age group are summarized in Table 2.

### 3.2. Children (from Weaning to 11 Years)

In children, several papers reported favism patients of Italian descent. 

One of the first reported cases of favism was in 1947, described by Wharton HJ and Duesselmann W [13]. The patient was a 3.5-year-old male child, who was admitted to the hospital for jaundice and dark urine for 16 h. The patient’s mother referred to the fact that on the day of the onset of the symptoms, the child had a large meal of fava beans, and that no such episode had ever happened to any of the family members. The physical examination described malaise, AHA, jaundice, pale mucous membranes and conjunctivae, and hepatomegaly. The laboratory examinations showed hemoglobin 4.2 g/dL, erythrocytes 1,140,000/mm^3^, and reticulocytes 4.2%; while, urine tests were positive for urobilinogen, bile, and albumin. In the following days, several transfusions were administered which led to a consistent change in hematochemical values and thus a rapid improvement in general condition. The diagnosis was made based on the history of fava bean consumption and on the laboratory findings. The patient was discharged on the eighth day without jaundice or anemia.

Rosen Ap and Scanlan JJ [14] reported in 1948 the case of a 5-year-old boy of Italian origins who had been hospitalized with abdominal pain, dark urine, and jaundice for 30 h. Two years earlier, the child had experienced the same symptoms, which resolved spontaneously without medication within two weeks. The patient was treated with a transfusion of whole blood and 5% glucose in water and saline. Following treatment, the clinical picture gradually improved. The onset of symptoms was traced back to the intake of four large plates of broad beans the previous day; also, the boy had eaten fava beans two years before, when the previous jaundice attack occurred. The diagnosis of favism was made by the authors based on the history of attacks following fava bean ingestion, as no fava bean sensitivity tests were available at the time. Additionally, the mother refused permission to give the child some fava beans to stimulate a reaction.

Similarly, in the case reported by Pickering DE and Hurwitz S [15] in 1951, there was no test available to investigate G6PD deficiency. The patient was a 28-month-old male child of Italian origin. On physical examination, he presented with jaundice, malaise, irritability, fever, vomiting, dark urine, yellowish sclerae, pale membranes, and a systolic murmur. The onset of symptoms was associated with the consumption of broad beans 36 h earlier. Treatment consisted of a whole blood transfusion, followed by an immediate improvement in condition; hemolysis stopped approximately 48 h after the onset of jaundice. The authors tried to use scratch tests with antigens prepared from fresh broad beans to prove the diagnosis, based on fava bean consumption, signs, and symptoms, but the result was negative.

In 1953, Larkin VD [16] reported the case of a 1-year-old girl of Italian origin with a one-month history of pallor in the eyes, ears, and lips following the ingestion of fava beans for two or three consecutive days. Laboratory findings showed hemoglobin 6.8 g/dL, erythrocytes 3,460,000/mm^3^, and a negative Coombs test. The patient was treated with vitamin B12, folate, and iron, and her color improved rapidly within a week. She was diagnosed with favism based on signs and symptoms.

In the same year, Diggle JH [18] reported two cases with different degrees of symptomatology in children of Cypriot origin. The first case was a 3.5-year-old male child admitted 30 h after the onset of symptoms, such as dark urine, abdominal pain, pallor, and jaundice. Physical examination revealed AHA, jaundice, and systolic murmur, and laboratory findings showed hemoglobin of 4.35 g/dL and a negative Coombs test. On spectroscopy examination, methemoglobin was found. Treatment consisted of whole blood transfusions, which led to improvement and discharge on the eighth day of hospitalization. The symptoms were associated with the ingestion of a plate full of broad beans on the day of the onset of the symptoms; based on this, and on the presence of typical signs and symptoms, the diagnosis of favism was made. Other cases of anemia were not reported in the mother’s, nor in the father’s family. The second case was a 9.5-year-old child admitted to the hospital with jaundice and anemia because of eating broad beans. The parents reported that he had a similar a symptomatological picture two years earlier. On admission, the physical examination revealed moderate jaundice, malaise, vomiting, and anemia; the hematochemical examination showed a hemoglobin of 9.9 g/dL and an erythrocyte count of 2,320,000/mm^3^, reticulocytes 8%, a serum bilirubin content of 6.8 mg/dL, and a negative Coombs test. No transfusion was necessary, and the clinical picture resolved spontaneously within four weeks. The authors prescribed an ounce of cooked broad beans for the patient, but this did not affect the hemoglobin level, so this association seems inconclusive. 

A few years later, in 1957, Tolmas HC [17] reported the case of a 2.5-year-old Italian male child admitted to the hospital for dark urine, jaundice, and pallor following the ingestion of broad beans two days earlier. The family history revealed that his maternal great-grandfather and paternal uncle also manifested such symptoms following the ingestion of broad beans. The diagnosis was made based on family history, signs, and symptoms. The patient was treated with dextrose in distilled water and a whole blood transfusion, as well as hydrocortisone, ascorbic acid, vitamin K, penicillin, and streptomycin. The patient’s condition improved continuously over the following days, and he was discharged on day three in good general condition.

Three papers analyze the cases of patients who have been hospitalized multiple times for symptoms developed after the ingestion of fava beans. Firstly, in 1955, McCarthy OR [19] reported the most severe exacerbation of favism in a 9-year-old British child who had several previous episodes of jaundice, always preceded by a fava bean meal. He was hospitalized for jaundice that appeared the same day he ingested fava beans, with shivers, malaise, and vomiting. The family history reported that the brother of the child’s mother also used to have jaundice and malaise whenever he ate broad beans. On physical examination, he had malaise, vomiting, jaundice, yellowish sclerae, and hepatomegaly. Laboratory tests are shown in Table 2. The jaundice resolved within 24 h and there were no longer other signs or symptoms, so the child was discharged with a diagnosis of favism and with the warning never to eat broad beans again. Then, in 1958, Brooks EA et al. [20] reported the case of a 3-year-old female child with abdominal pain, vomiting, and dark urine, pallor, coryza, and jaundice. The patient was treated with a whole blood transfusion, which led to a general improvement in clinical condition and hematochemical parameters. The patient was then readmitted to the hospital five months after the first episode with the same symptoms, although less pronounced than before. This second episode was resolved in 10 days, with no need for transfusion. The patient was discharged after the parents were warned not to let the child eat broad beans anymore. The author found it interesting that the osmotic fragility of the red blood cells of the patient’s father, and sister, slightly decreased. It should be noted that this article was written in 1958 when favism had not yet been discovered. Finally, in 1960, Gower ND and Frommer E [21] reported the case of a 3-year-old Cypriot male child admitted to the hospital with dark urine and pallor for 24 h, and several episodes of vomiting, pallor, malaise, jaundice, enlarged tonsils and lymph nodes. Treatment consisted of a transfusion of packed red blood cells, as well as cortisone, prednisolone, and penicillin. Following treatment, the patient’s clinical condition improved rapidly, and he was discharged on day 11 of hospitalization. The patient was seen later after a few days of sore throat and one day of dark urine, following the ingestion of broad beans the day before, but recovery was rapid and did not require a transfusion. At first, the author ruled out a diagnosis of mild congenital hemolytic anemia, and even after the second hospitalization, investigations revealed no red blood cell defects; however, after full recovery, the authors were able to demonstrate that the red blood cells behaved abnormally after incubation with acetylphenylhydrazine, and thus a diagnosis of favism was made. 

Discombe G and Mestitz W [22] reported, in 1956, the case of a 2.5-year-old girl of Middle-Eastern descent with a history of 1–2 days of fever, malaise, and yellowish eyes. The patient was treated with a transfusion of packed red cells, followed by continuous improvement until complete remission on day seven. Symptoms started after she ingested fava beans either 1–2 days before and on the same day. The authors emphasize that there was no evidence that this was a favic attack and that it would be unethical to try to prove this by inducing it.

Two particular cases of severe ophthalmological complications following favism were reported by Choremis C et al. [23] in 1960. The first case was a 3-year-old boy admitted to the hospital for malaise, vomiting, dark urine, and jaundice after eating broad beans the day before. On admission, the patient was comatose, with spastic legs, a plantar reflex in extension, hepatomegaly, and splenomegaly. Laboratory findings showed hemoglobin 6 g/dL, erythrocytes 2,300,000/mm^3^, serum bilirubin 5.8 mg/dL, and indirect bilirubin 2.0 mg/dL. Treatment consisted of several daily transfusions of whole blood. On the third day of admission, the general condition worsened with the appearance of ecchymoses, but in the following days, the patient came out of the coma and the hematochemical parameters improved, but he was completely blind. On ophthalmological examination, the pupils were mydriatic and unresponsive to light; there were diffuse retinal and pre-retinal hemorrhages in the posterior pole and in the hyaloid body, explained by the authors as a consequence of the generalized hemorrhagic state due to favism. The prognosis was poor due to the hemorrhages in the hyaloid body. The second case was a 6-year-old boy admitted to the hospital for blindness, which developed progressively two days after an attack of favism, and apparently resolved without treatment. On admission, the results of the physical examination and laboratory tests were essentially normal. On ophthalmological examination, edema and blurring of the disc margins, as well as constriction of the retinal vessels with small hemorrhages, were found, which, together with the sudden bilateral loss of vision, might be associated with the acute hemolytic syndrome that had occurred 7 days earlier due to favism. The authors reported that post-hemolytic anemia, together with a trigger factor (the one responsible for the hemolysis), may have caused severe ocular involvement.

In 1966, Stewart AG et al. [24] reported the first case of favism in Canada. A 4-year-old Norwegian-Ukrainian male complained of malaise, abdominal pain, anorexia, dark urine, and several episodes of vomit. The physical examination showed a semi-comatose child with jaundice, pallor, and yellowish eyes. The treatment consisted of a whole blood transfusion, intravenous fluids, and methylprednisolone. The patient was oliguric until the third day of hospitalization when diuresis started to improve rapidly. After one week, he was discharged in good condition. The onset of symptoms occurred one day after a meal of broad beans, cooked and raw. This was not the first time the child had eaten broad beans, but it is not clear whether previous ingestions were followed by the disease. Five months after the episode, the G6PD activity was found to be 0 U/g Hb.

Some years later, in 1989, Wong WY et al. [25] reported the case of a 2.5-year-old male child who presented with dark urine, pallor, and a reduced appetite. On physical examination, no relevant parameters were found, other than a grade 1/6 systolic murmur. Red cell transfusion resulted in complete remission in four days. Because of the language barrier, the authors report that the parents initially denied ingesting fava beans, but later reported that the child had eaten a common Asian snack made from seed pods of the fava plant. A laboratory test held several months later showed a 0 IU/g Hb of G6PD enzyme activity (normal range, 7.9 to 16.3 IU/g Hb).

Galiano S et al. [26] reported three cases of favic attacks due to variant A^−^ in 1990. The first case was of a 19-month-old Italian male child admitted for dark urine and malaise, three days after the ingestion of broad beans. Four months later, G6PD activity was 10% of the normal value. The second case was of a 4-year-old black male admitted to the pediatric clinic due to the appearance of dark urine, anemia, vomiting, and headache, 60 h after ingesting beans; the laboratory tests revealed a hemoglobin concentration of 5.1 g/dL and reticulocytosis of 9%. Investigations on the activity of the G6PD reported a value equal to 12% of normal. The latest reported case was a six-month-old Anglo-Jamaican girl admitted to the hospital for jaundice, paleness, and dark urine, 24 h after the first ingestion of broad beans; she also ingested them on the day of admission. Her father was found to have 7% of normal G6PD enzyme activity, so the authors concluded that he was heterozygous for G6PD deficiency, and diagnosed favism. No data were reported on the treatment used.

More recently, in 2011, Odièvre MH et al. [27] reported the case of a 6-year-old Algerian male presenting with symptomatic methemoglobinemia, meningitis, fever, headache, vomit, cyanosis of the lips, and 80% oxygen saturation, resistant to oxygen therapy. The day after admission, the patient was pale, tachycardic, and passed dark urine, while hemoglobin had dropped to 6.0 g/dL and the methemoglobin fraction had increased to 8.7%. Treatment consisted of an oxygen mask and a transfusion of packed red blood cells. Upon questioning, it was discovered that the child had eaten broad beans the day before, and that there was a family history of G6PD deficiency. A laboratory examination of the child’s blood revealed that he was hemizygous for variant A- with an enzyme activity of 6 U/g Hb (normal range: 8–22 U/g Hb).

Additionally, Leunbach TL et al. [28] reported in 2014 two cases of methemoglobinemia. The first was a 4-year-old Iraqi male admitted with fever, fatigue, AHA, jaundice, and cyanosis with SpO2 74%, which developed two days after fava bean ingestion. Oxygen therapy followed by a transfusion made the cyanosis disappear and raised the SpO2 to 85%. The hemolytic episode resolved without any problems and G6PD deficiency was demonstrated to be 0.08 kU/mol (normal value: 0.51–1.32 kU/mol). The second case reported was a 6-year-old Iraqi male without cyanosis, but with SpO2 at 78%, resistant to supplemental oxygen, and AHA, which developed the day after the ingestion of broad beans. Again, a packed red blood cell transfusion was performed, which led to an improvement in SpO2 and all chemical parameters over the next two days. In this case, favism was diagnosed with a G6PD activity <0.10 kU/mol (normal value: 0.51–1.32 kU/mol).

In 2013, Mohamed M and Els I [29] reported the case of a 15-month-old Chinese male child admitted to the emergency room for malaise, jaundice, paleness, and yellow sclera, which lasted for 24 h following the ingestion of bean soup. The child had hemizygous G6PD deficiency, with an assay of 3.2 IU/g Hb, lower with respect to the normal range (8.8–17.9 IU/g Hb).

One year later, in 2014, Verdugo LP et al. [30] described the severe case of a 31-month-old male child admitted to the pediatric critical care unit for malaise, abdominal pain, paleness, fever, and vomiting. Upon admission, laboratory tests showed AHA: hemoglobin 4.7 g/dL, hematocrit 15.8%, erythrocytes 2,000,000/mm^3^, reticulocytes 4.4%, total bilirubin 4.11 mg/dL and indirect 3.53 mg/dL, Coombs test negative. The manifestation of symptoms occurred 48 h after the ingestion of fresh broad beans. The treatment consisted of a transfusion of packed red blood cells, intravenous hydration, and folic acid, which led to a progressive improvement in the laboratory tests and therefore the clinical condition. A Toenz and Bretke test confirmed the G6PD deficiency.

In the same year, Zuccotti GV et al. [31] reported the case of an 8-month-old male child, weaned, who developed jaundice due to favism. The examination revealed hemoglobin 7.6 g/dL, erythrocytes 2,500,000/mm^3^, hematocrit 23%, reticulocytes 3.7%, total bilirubin 10.6 mg/dL and unconjugated 10.3 mg/dL; while, a second sample revealed an even greater AHA: hemoglobin 5.6 mg/dL, hematocrit 17.7%, LDH 739U/L, and Coombs test was negative. The child was treated with a packed red cell transfusion. A laboratory examination revealed a G6PD activity value of 16 U/1012 RBC (reference ranges: 146–376 U/1012 RBC). The authors speculated that the cause of AHA was cross-contaminated pumpkin seeds with broad beans. The contamination was proven when fava bean DNA was found in the frozen pumpkin DNA, extracted from the sample eaten by the child.

Wadowski B et al. [32] reported, in 2016, the case of a 2-year-old Chinese male child admitted to the hospital with paleness, malaise, and dark urine, as well as a one-week history of intermittent fever. The patient was treated with multiple packed red cell transfusions, which resulted in the resolution of the symptoms. Thanks to an interpreter, it was possible to identify that the child ate a cup of broad beans and drank traditional Chinese tea, one day before the onset of symptoms, and a test confirmed the G6PD deficiency. 

The spectrum of clinical features in this age category presents a wide variety: systolic murmur, semi-coma or coma state, methemoglobinemia, and partial or total loss of vision, in addition to the classic symptoms. The time elapsed between the intake of fava beans and the onset of symptoms varies from 1 to 3 days. Only two articles reported the G6PD variant (i.e., A^−^), which falls in class B according to the WHO classification [1], corresponding to a median activity of less than 45% of normal and acute or triggered hemolysis. In other cases, the variant is not described. Due to the lack of data and the large number of variants of the allele discovered up to now, we cannot associate symptoms with a specific variant. In three reports, patients were hospitalized twice for fava bean ingestion, while in all other reports it is not mentioned whether the children had further attacks. All the characteristics of this age group are summarized in Table 2.

### 3.3. Pre- and Adolescents (11–18 Years)

In 1955, Mansoor D. [33] reported two cases of favism in Israel. The first case was a 12-year-old male admitted in a semi-comatose state, with no plantar response but eye and tendon reflexes present. On physical examination, the patient presented with dark urine, pallor, hepatomegaly, limbs with spasmodic contractions, and profuse sweating. The symptoms were associated with the ingestion of broad beans for two consecutive weeks, which together with the presence of typical signs and symptoms, led to the diagnosis of favism. The patient was treated with several transfusions, followed by an improvement in hemoglobin from 3.0 g/dL on day 1 to 7.0 g/dL after the second transfusion. He was discharged on the 8th day, after an uneventful recovery. As in the former case, the latter was a 12-year-old male, with symptoms triggered by the ingestion of broad beans. As the patient had a hemoglobin of 2.0 g/dL, he was treated with two transfusions and discharged after two weeks with a diagnosis of favism. 

Holt JM and Sladden RA [34] reported, in 1965, two cases, one which might have proved fatal. The first case was a 14-year-old male of Greek origins admitted to the hospital for extreme tiredness, noises in the ears, a fainting episode, several episodes of vomit, malaise, jaundice, weakness, dark urine, systolic murmur, extensor plantar responses, and semi-coma. The G6PD level was 0.4 units (normal range 2.1–4.2 units), and symptoms followed the ingestion of a large quantity of broad beans, eaten daily for a week; a diagnosis of favism was therefore made. The treatment consisted of a whole blood transfusion, which led to an improvement in general condition. The second case was a 10-year-old Italian male admitted to the hospital for epigastric pain and malaise. On physical examination, he had malaise, yellowish sclerae, dark urine, and hepatomegaly. The erythrocyte G6PD level was found to be 63 units (normal range: 150–217 units) and so the diagnosis was confirmed. After that, the patient was discharged on the sixth day of recovery when his hemoglobin level was raised to 9.9 g/dL without any treatment. Upon questioning, it turned out that broad beans were a regular part of the family’s diet and that he had never had any effects, but this time, for the first time, the child had eaten them raw. 

Oliveira S et al. [35] reported, in 2000, the case of a 16-year-old European-Caucasian male with dark urine, fever, and abdominal pain that developed two days after ingesting broad beans for the first time. On physical examination, jaundice, pallor, hepatomegaly and splenomegaly, and a painful hypogastric region were present. Blood examination revealed AHA, unconjugated hyperbilirubinemia, elevated LDH, bilirubinuria, and hemoglobinuria. On family history, the mother reported that she had also had a similar episode due to the ingestion of broad beans many years earlier. As the child refused a blood transfusion, the only treatment was intravenous hydration. During the first two days, there was a drop in hemoglobin, but on the third day (the fifth after the beans were ingested), the laboratory parameters started to improve. Laboratory tests on the patient’s blood and that of his family members revealed the presence of a G6PD deficiency in the patient himself (0.92 IU/g Hb; normal range: 5–7.1 IU/g Hb), his mother, and two of his brothers.

In 2006, Lau HK et al. [36] published a retrospective review of six Hong Kong male patients admitted to the Department of Pediatrics of the Tuen Men Hospital from March 1993 to February 2005, with acute massive hemolysis with a neonatal diagnosis of G6PD deficiency. Two of them had experienced a hemolytic crisis after the ingestion of fava beans, while the other four developed symptoms after exposure to mothballs (n = 1), treatment with herbal medicine or intramuscular injection of unknown nature (n = 2), and upper respiratory tract infection (n = 1). The first one was an 11.3-year-old, and the second one was an 8.8-year-old. Both of them were admitted to the pediatric intensive care unit and an erythrocyte transfusion was performed (5 units and 1 unit, respectively). They all made a complete recovery. 

The severity of symptoms in this age category varies from near-fatal episodes, probably due to the large quantity of fava beans ingested, to very mild symptoms that resolved spontaneously. The time elapsed between fava bean ingestion and the onset of symptoms varies within a narrow range of 1 or 2 days; in two cases, the onset of the hemolytic attack followed after a week or more of consecutive fava bean ingestion. In only one case the degree of deficiency was detected. All the clinical features of this age group are summarized in Table 2.

### 3.4. Adults (>18 Years)

We found some cases of favism in adulthood from 34 years old to 74 years old. 

Stockley R et al. [37] first reported favism in adults in 1985. The authors described two cases of women with attacks of favism due to fava bean intake, two days before and one day before symptom onset, respectively. The first woman, aged 56, had eaten fava beans several times before, but never as many as this time; she recalled an episode of AHA and jaundice 10 years earlier, as well as having had jaundice as a child. Laboratory tests showed a hemoglobin level of 10 g/dL and serum bilirubin of 7.84 mg/dL, while G6PD enzyme activity was found to be low at 3.7 IU/g Hb (normal range: 4.6–13.5). Treatment consisted of packed red blood cell transfusion and hydration, which resulted in the resolution of jaundice in four days. The second woman, aged 41, presented with a 24 h history of jaundice and dark urine; in this case, it is not reported whether fava beans had ever been ingested before, or whether these symptoms had been present in the past. Laboratory tests showed a hemoglobin level of 9.6 g/dL and serum bilirubin of 3.8 mg/dL, and a negative Coombs test. The G6PD enzyme level was reduced to 2.1 IU/g Hb, but after two weeks was found to be 3.4 IU/g Hb. Jaundice and general clinical conditions resolved spontaneously.

Hasler J and Lee S [38] reported, in 1993, the case of a 46-year-old Middle-Eastern man with a history of several days of malaise, intermittent fever, persistent nausea, and vomiting, as well as myalgia and arthralgia of the left shoulder and lower spine, due to the ingestion of a large amount of raw fava beans, six to eight cocktails, and many glasses of wine at a party the day before the onset of symptoms. On physical examination, the patient presented jaundice, mild distress, dark and intermittently bloody urine, yellowish eyes, and sinus tachycardia without murmur. Laboratory results showed hemoglobin 5.4 g/dL, hematocrit 15.8%, reticulocytes 21%, total bilirubin 7.5 mg/dL, LDH 1682 U/L. He was treated with oxygen supplementation, intravenous normal saline, and a whole blood transfusion. Recovery was not difficult, and the patient was discharged in good general condition with a diagnosis of favism, based on typical signs and symptoms.

In 1997, Hampl JS et al. [39] reported the case of a 34-year-old Iraqi male who developed AHA, renal failure, and jaundice the day after eating broad beans. The patient took Vick Formula 44 to treat an episode of vomiting, fever, and anorexia, which occurred four days before admission. Physical examination revealed dark urine and yellowish eyes, and a dry mouth. Following laboratory results (hemoglobin: 3.6 mg/dL, erythrocytes: 900,000/mm^3^, hematocrit: 9%, serum bilirubin: 2.98 mg/dL) and analysis of the G6PD enzyme level, which was 2.6 U/g Hb (normal range: 4.6–13.5 U/g Hb), the patient was diagnosed with favism. The patient was treated with hemodialysis and transfusions of packed red blood cells, in addition to oral folate and vitamin supplements. He continued hemodialysis for 2 weeks after his discharge. 

Lim F et al. [40] reported, in 2005, the case of a 44-year-old female who presented with malaise, vomiting, yellowish eyes, and dark urine after consuming two meals of boiled fava beans, two days before the onset of symptoms and even on the same day. Laboratory findings showed anemia and reticulocytosis. Physical examination showed jaundice and ankle edema. The patient was treated with a whole blood transfusion. The family history showed no cases of jaundice or AHA, but during the laboratory investigation for the analysis of G6PD enzyme activity, she was found to be heterozygous for the 563 C > T mutation of the Mediterranean variant, with an activity of 2.5 U/g Hb (normal range: 4.6–13.5 U/g Hb).

More recently, in 2011, Soyuncu S et al. [41] reported the case of a 56-year-old man with a 2 day history of yellowish eyes, dizziness, and recurrent attacks of syncope, hemoglobin 8.4 g/dL and hematocrit 23.7%. The G6PD level was low and, although it was not the first time the patient had eaten fava beans, in this case, it caused an exacerbation of the reported symptoms. Treatment consisted of a transfusion of packed red blood cells, which improved the general clinical condition.

In 2021, two articles were published reporting favism attacks in adults. Ata F et al. [42] reported the case of a 56-year-old Qatari man with dyspnea, dizziness, and vomit that developed after a large meal of fava beans—a food that had been consumed previously, although in smaller quantities. On physical examination, he presented with pallor, jaundice, low oxygen saturation, resistance to supplementation, and a methemoglobin level of 5.6%, for which he received intravenous (IV) methylene blue 80 mg. Laboratory tests revealed a hemoglobin level of 9.9 gm/dL, which decreased to 7 g/dL after 24 h. He was treated with a red blood cell transfusion, which did not stop the drop in hemoglobin. He was diagnosed with G6PD deficiency (23 mU/10^9^ RBC, normal value: 224–517 mU/10^9^ RBC), and was discharged as he became asymptomatic on the fifth day. Al-Dubai H et al. [43] reported the case of a 47-year-old man with a 3 day history of yellowish eyes and dark urine, arising two days after a medium-sized plate of broad beans. On physical examination, there was jaundice, methemoglobin level of 3.6%, and a decreased oxygen saturation, which was resistant to supplementation. G6PD activity was found to be low (24 mU/10^9^ RBC, normal value: 191–327 mU/10^9^ RBC), and so a diagnosis of G6PD deficiency was made. The treatment consisted of paracetamol and supplemental oxygen, but no transfusion was required. He was discharged after two days, after being counseled on food and drugs to avoid.

We found only two cases in which the attack of favism developed in elderly age: in the first case, the patient had already had two similar episodes, 12 years and 6 years earlier, and developed the classic symptoms that were resolved using whole blood transfusions; while in the second case, the patient had never had previous episodes and the attack of hemolysis led to severe renal failure. The clinical aspects are discussed in more detail below. 

In 1959, Hartigan JD and Gurnett TJ [44] reported the case of a 74-year-old male, who developed dark urine, weakness, jaundice, malaise, and fever, after a dinner of broad beans. He had experienced two previous episodes with such symptoms, which led to the diagnosis of favism. 

In 2012, Torres C D et al. [45] reported the case of a 67-year-old man with a 2 day history of abdominal pain, jaundice, choluria, and malaise that developed after eating beans. Physical examination showed oliguric acute renal failure, requiring dialysis support (see Table 2). Three months after discharge, G6PD activity was investigated with a Toenz and Betke test, which showed undetectable enzyme activity. There was no family history of favism. 

To summarize, in adults (Table 2), the scenarios reported include the following:

1. Adults who eat broad beans for the first time in adulthood and develop symptoms associated with favism.
2. Adults who have eaten broad beans before but without any symptoms associated with favism, which, on the other hand, are now present in the reported case. In this case, exacerbation of symptoms is usually associated with a much higher fava bean intake than the dose normally taken.
3. Adults who have eaten broad beans and have previously experienced symptoms associated with favism.

Moreover, the spectrum of symptoms includes renal failure, of different degrees, in addition to the classic ones. Treatment can also vary widely: from requiring several transfusions and hemodialysis, to being self-limiting and resolving on its own. One article reported the G6PD variant (i.e., Mediterranean), with a 563 C > T mutation, which again falls into class B, just like the A^-^ variant previously described; while in a few cases, the authors investigated the G6PD activity, which was always found to be low or absent.

## 4. Conclusions

Age of onset seems to play an important role in clinical presentation, but it is not certain whether this exceeds the severity of the enzymatic deficiency itself. The erratic feature of the gene is the factor responsible for the wide variability in symptom exacerbation. As reported in several cases, the onset of symptoms is not always concomitant with the first ever fava bean ingestion, but it is also true that it usually appears when the amount of fava beans ingested is greater than the amount previously eaten, or when fava beans are eaten continuously for several days. This is especially evident in youngest children or pregnant women when the severity of the symptoms could be explained by the unbalanced ratio between volume of beans ingested and the patient’s body mass.

The clinical picture described has always an underlying state of acute hemolytic anemia, which is one of the most specific symptoms of favism. The laboratory findings are characterized by anemia, with a very wide range of hemoglobin (2.0–12.0 mg/dL), reticulocytosis (from 2% up to 65%), which stands for an increased bone marrow activity trying to compensate the hemolysis, elevated unconjugated bilirubin level, and sometimes by the presence of urinary urobilinogen and of methemoglobinemia.

The symptomatology spectrum encloses specific, aspecific and rare symptoms. The specific symptoms for favic attacks were jaundice, developed due to the increased bilirubin following increased erythrocyte depletion, and therefore splenomegaly, sometimes even hepatomegaly, discolored urine, tachycardia, and pallor. The aspecific symptoms were abdominal pain, malaise, vomiting, nausea, and dizziness. From our review, we found an association between rare symptoms and age of onset:

- two cases of death were reported, probably due to the high ratio between quantity of fava beans ingested and the weight of the patient (infants);
- two cases of sight problems and blindness were described as a complication of the favic attack, both in children;
- several cases of systolic murmur were observed in infants, children, and adolescents;
- three cases of renal failure were found, in adults, to be associated with favism.

With the fatal exception of death in infants, the symptomatology is self-limited and does not release sequelae, but in most cases hospitalization and transfusion, of whole blood or packed red blood cells, are required.

When favism is diagnosed, it is more common to detect the severity of the deficit than the detection of the variant itself. Nevertheless, some considerations can be made. In the reported cases, the Gd gene variants can be categorized into class B, corresponding to cases in which a hemolytic crisis developed, and class C, for those less severe cases in which hemolytic anemia was not present.

The type of fava beans eaten also plays a role in the likelihood of symptom onset: raw fava beans are more likely to induce the crisis, than cooked, frozen, or canned ones; in addition, the amount of glucosides is directly proportional to degree of ripeness, so the more unripe the fava beans, the less the amount of vicine and convicine, and thus the lower the risk of the crisis onset. Despite several professional and patient associations educating G6PD deficient patients to avoid all kinds of beans and pulses and several other medications or products [47], no consideration can be made from the results of the present review. Nonetheless, we feel that further studies should better investigate the effect of the consumption of pulses other that fava beans on G6PD deficient patients with different enzymatic variants.

As for fava bean consumption, available evidence suggests the following: if the enzyme deficiency is known, the best strategy to avoid the onset of a favic crisis is the complete elimination of fava beans from one’s diet; on the other hand, if the deficiency is not known, due to lack of family history of anemia, jaundice, or other symptoms that may resemble favism, some precautions can be taken: avoid very large fava bean meals, preferably consume fava beans that are not too ripe, and choose frozen, boiled, or canned versions; however, none of these precautions give a complete guarantee of the absence of danger.

Finally, a small mention must be made of the importance of avoiding cross-contamination; in fact, a case described the harmful exacerbation of symptoms due to the ingestion of pumpkin seeds contaminated with broad beans. Therefore, in the presence of G6PD deficiency carriers, it is necessary to pay particular attention to the preparation of foods and their storage: avoid using dirty utensils and dishes previously used for cooking and/or preparing broad beans, store the beans in cupboards in special containers that prevent contact with other foods, check the labels of ready-made foods, and in the case of meals in a restaurant, communicate the condition to warn of the need for precaution.

## Figures and Tables

**Table 1:** WHO classification of G6PD variants.

Class	Median of G6PD Activity	Hemolysis
A	<20%	Chronic
B	<45%	Acute, triggered
C	60–150%	No hemolysis
U	Any	Uncertain clinical significance

**Table 2:** Clinical features of favism in different age groups.

	Age (Years Old)	Gender	Triggering Foods	Timing (n. Days)	Previous Episodes	First Ingestion	Family History	Ethnicity	Signs & Symptoms	Vital Parameters	Hb (g/dL)	Erythrocytes (x mm^3^)	Ht (%)	Reticulocytes (%)	Serum Bilirubin (mg/dL)	Urinary Urobilinogen	Coombs Test	Transfusion	G6PD Test
Clinical features of favism in pregnant women and infants																			
Cahill KM and Lay AB [7]	35 (pregnant)	F	Fresh fava beans	1–2	No	No	Yes	Greek	Dark urine Hepatomegaly Jaundice Malaise Splenomegaly Systolic murmur	NR	6.0	3,000,000	20	7.6	Total: 3.8 Indirect: 2.9	Positive	Negative	No	Yes (8.9 U/g Hb)
Mentzer WC and Collier E [8]	Newborn	M	Fried and then dried fava beans	Last month of pregnancy	No	NR	NR	NR	Death	NR	9.8	NR	29	NR	NR	NR	Negative	NR	Yes
Corchia C et al [9]	2 h old	F	Cooked dried fava beans	5	No	NR	NR	NR	Hepatomegaly Pallor Splenomegaly Systolic murmur	BP: 52 mmHg	7.3	1,900,000	23	9	10.2	NR	Negative	Yes (whole blood)	Yes (4.61 U/g Hb)
Casper J and Shulman J [10]	6 months old	M	Fava beans	2	No	Don’t know	No	Yemenite	Death	NR	5.4	1,500,000	NR	65	NR	NR	NR	Yes (whole blood)	NR
Emanuel B and Schoenfeld A [11]	4 months old	F	Fava beans	1	No	No	No	Egyptian	Dark urine Hepatomegaly Jaundice Malaise Pallor Splenomegaly	BT: 36.8 °C HR: 180 bpm RR: 60/min	5.7	1,800,000	NR	8.8	Direct: 0.9 Indirect: 3.4	Positive	Negative	Yes (whole blood)	Yes
Taj-Eldin S [12]	4 months old	M	Boiled dried fava beans	4	No	NR	NR	Arabic	Dark urine Hepatomegaly Jaundice Malaise Pallor	BT: 37.2 °C HR: 160 bpm RR: 50/min	3.8	1,480,000	NR	7	Total: 4.2 Indirect: 3.6	Positive	NR	Yes (whole blood)	Yes
	2 months old	M	Fava beans	5	No	NR	NR	Arabic	Dark urine Hepatomegaly Jaundice Pallor	NR	4.7	2,100,000	NR	6	Total: 3.8	Positive	NR	NR	Yes
	4 months old	M	Fava beans	5	No	NR	NR	Arabic	Dark urine Jaundice Pallor Splenomegaly	NR	5.2	1,900,000	NR	12	Total: 4.5 Indirect: 3.2	Positive	NR	NR	Yes
	3 months old	M	Fava beans	4	No	NR	NR	Arabic	Dark urine Hepatomegaly Jaundice Pallor	NR	4.0	1,210,000	NR	10	Total: 5.0 Indirect: 3.5	Positive	NR	NR	Yes
Clinical features of favism in children																			
Wharton HJ and Duesselman W [13]	3.5	M	Fava beans	0	NR	NR	No	Italian	Anemia Dark urine Hepatomegaly Jaundice Malaise Pallor	BT: 102 °F HR: 160 bpm RR: 30/min	4.2	1,140,000	NR	4.2	NR	Positive	NR	Yes (whole blood)	NR
Rosen AP and Scanlan JJ [14]	5	M	Fava beans	1	Yes	No	NR	Italian	Abdominal pain Dark urine Jaundice Pallor Splenomegaly Systolic murmur Vomit	BT: 101.6 °F BP: 98/68 mmHg HR: 30 bpm	5.5	1,520,000	35	3.8	NR	NR	NR	Yes (whole blood)	NR
Pickering DE and Hurwitz S [15]	28 month old	M	Fava beans	1–2	NR	No	NR	Italian	Dark urine Jaundice Malaise Systolic murmur	NR	5.75	2,500,000	18	NR	NR	Positive	Negative	Yes (whole blood)	NR
Larkin VD [16]	1	F	Fava beans	NR	NR	NR	NR	Italian	Pallor	NR	6.8	3,460,000	NR	NR	NR	NR	NR	No	NR
Tolmas HC [17]	2.5	M	Fava beans	2	NR	NR	Yes	Italian	Dark urine Hepatomegaly Jaundice Pallor Splenomegaly Systolic murmur	BT: 101 °F BP: 100/70 mmHg HR: 140 bpm	5.4	1,910,000	17	8.8	Direct: 0.3 Indirect: 2.6	NR	Negative	Yes (whole blood)	NR
Diggle JH [18]	3.5	M	Fava beans	NR	NR	No	NR	Cypriot	Abdominal pain Anemia Dark urine Jaundice Pallor Systolic murmur	NR	4.35	NR	NR	NR	NR	NR	Negative	Yes (whole blood)	NR
	9.5	M	Fava beans	0	NR	NR	NR	Cypriot	Anemia Jaundice Malaise	NR	9.9	2,320,000	NR	8	6.8	Negative	Negative	No	NR
McCarthy OR [19]	9	M	Cooked homegrown fava beans	2	No	NR	Yes	NR	Hepatomegaly Jaundice Malaise Vomit	NR	NR	3,400,000	NR	24	0.3	NR	Negative	NR	NR
Brooks EA et al. [20]	3	F	Raw fava beans	0-2	Yes	No	NR	NR	Abdominal pain Dark urine Jaundice Pallor Vomit	NR	3	850,000	NR	8	3	Positive	Negative	Yes (whole blood)	NR
	3.5		Raw fava beans	2	Yes	No			Abdominal pain Dark urine Jaundice	NR	7.5	2,500,000	NR	1	4.5	Positive	Negative	No	NR
Gower ND and Frommer E [21]	3	M	Fava beans	NR	No	Yes	NR	Cypriot	Dark urine Malaise Jaundice Pallor Vomit	BT: 100 °F HR: 132 bpm RR: 28/min	3.6	NR	NR	NR	4	NR	Negative	Yes (whole blood)	Yes
	4		Frozen fava beans	1	Yes	No	NR		Dark urine Sore throat	NR	NR	NR	NR	NR	NR	NR	NR	No	Yes
Discombe G and Mestitz W [22]	2.5	F	Fava beans	0-2	NR	NR	No	Middle-eastern	Malaise	NR	4.4	1,500,000	NR	NR	NR	Positive	NR	Yes (packed RBC)	NR
Choremis C et al. [23]	3	M	Fava beans	1	NR	NR	NR	NR	Blindness Coma Dark urine Hepatomegaly Jaundice Malaise Splenomegaly Vomit	NR	6	2,300,000	NR	NR	Total: 5.8 Indirect: 2.0	NR	NR	Yes (whole blood)	NR
	6	M	Fava beans	NR	NR	NR	NR	NR	Partial loss of sight	NR	NR	NR	NR	NR	NR	NR	NR	No	NR
Stewart AG et al. [24]	4	M	Freshly harvested fava beans	1	Yes	No	NR	Norwegian Ukrainian	Abdominal pain Dark urine Jaundice Pallor Semicoma Vomit	NR	6.1	NR	16	9	Total: 6.9 Direct: 0.9	NR	Negative	Yes (whole blood)	Yes (0 U/g Hb)
Wong WY et al. [25]	2.5	M	Yewdow nuts	NR	NR	NR	NR	NR	Dark urine Jaundice Pallor Systolic murmur	BP: 107/47 mmHg HR: 104 bpm RR: 40/min	4.1	NR	11	6.8	Total: 6.6 Direct: 0.3	NR	Negative	Yes (packed RBC)	Yes (0 U/g Hb)
Galiano S et al. [26]	19 months old	M	Fava beans	3	NR	NR	NR	NR	Dark urine Malaise	NR	5.0	NR	NR	7	NR	NR	Negative	NR	Yes
	4	M	Fresh fava beans	2-3	NR	NR	NR	NR	Anemia Dark urine Vomit	NR	5.1	NR	NR	9	NR	NR	NR	NR	Yes
	6 months old	F	Fresh fava beans	1	NR	NR	NR	English-Jamaican	NR	NR	6.6	NR	NR	5	NR	NR	Negative	NR	Yes
Odièvre MH et al. [27]	6	M	Fava beans	1	NR	NR	Yes	Algerian	Dark urine Lips cyanosis Pallor Vomit	Sat02: 80%	9.2	NR	NR	NR	NR	NR	NR	Yes (packed RBCs)	Yes (6 U/g Hb)
Leunbach TL et al. [28]	4	M	Fava beans	2	NR	NR	NR	Iraqi	Anemia Cyanosis Jaundice Malaise	Sat02: 74%	9.0	NR	NR	NR	4.33	NR	NR	Yes (packed RBCs)	Yes (0.08 kU/mol)
	6	M	Fava beans	1	NR	NR	NR	Iraqi	Anemia	Sat02: 78%	6.77	NR	NR	NR	6.55	NR	NR	Yes (packed RBCs)	Yes (<0.10 kU/mol)
Mohamed M and Els I [29]	15 months old	M	Fava bean soup	1	No	NR	No	Chinese	Jaundice Pallor	NR	5.0	NR	NR	NR	NR	NR	NR	NR	Yes (3.2 U/g Hb)
Verdugo L P et al. [30]	2.7	M	Fresh fava beans	2	Yes	NR	NR	NR	Abdominal pain Anemia Hepatomegaly Jaundice Pallor Vomit	HR:156 bpm	4.7	2,000,000	15.8	4.4	Total: 4.1 Indirect: 3.5	NR	Negative	Yes (packed RBC)	Yes
Zuccotti GV et al. [31]	8 months old	M	Pumpkin seeds cross-contaminated with fava beans	1	NR	No	NR	NR	Jaundice	NR	7.6	2,500,000	23	3.7	Total: 10.6 Indirect: 10.3	NR	Negative	Yes (packed RBC)	Yes (16 U/1012 RBC)
Wadowski B et al. [32]	2	M	Fava beans and a traditional Chinese herbal tea	1	NR	NR	NR	Chinese	Dark urine Malaise Jaundice Pallor	Sat02: 88%	4.1	NR	11.8	7	Total: 8.1 Direct: 0	NR	Negative	Yes (packed RBC)	Yes
Clinical features of favism in pre- and adolescents																			
Mansoor S [33]	12	M	Fava beans	0-14	NR	NR	NR	Israeli	Dark urine Hepatomegaly Pallor Semicoma Vomit	BT: 38 °C BP: - HR:140 bpm	3.0	2,360,000	NR	NR	10	NR	NR	Yes (whole blood)	NR
	12	M	Fava beans	NR	NR	NR	NR	Israeli	NR	NR	2.0	NR	NR	NR	NR	NR	NR	Yes (whole blood)	NR
Holt Jm and Sladden RA [34]	14	M	Fava beans	0–7	NR	NR	NR	Greek	Dark urine Jaundice Malaise Systolic murmur Vomit	NR	4.3	NR	NR	29	7.0	NR	NR	Yes (whole blood)	Yes (0.4 U)
	10	M	Raw fava beans	NR	NR	No	NR	Italian	Abdominal pain Dark urine Hepatomegaly	NR	6.7	NR	NR	12	1.0	NR	NR	NR	Yes (63 U)
Oliveira S et al. [35]	16	M	Fava beans	2	NR	Yes	Yes	European-Caucasian	Abdominal pain Dark urine Hepatomegaly Jaundice Pallor Splenomegaly	NR	NR	NR	NR	NR	NR	Positive	NR	No	Yes (0.92 IU/g Hb)
Lau HK et al. [36]	11.3	M	Fava beans	NR	NR	NR	NR	Hong Kong	NR	NR	7.1	NR	NR	6,7	7.43	NR	NR	Yes (packed RBCs)	NR
	8.8	M	Fava beans	NR	NR	NR	NR	Hong Kong	NR	NR	4.4	NR	NR	14,0	6.96	NR	NR	Yes (packed RBCs)	NR
Clinical features of favism in adults																			
Stockley R et al. [37]	56	F	Fava beans	2	Yes	No	NR	NR	NR	NR	10	NR	NR	NR	7.84	NR	NR	Yes (packed RBCs)	Yes (3.7 IU/g Hb)
	41	F	Home-grown fava beans	1	NR	NR	NR	NR	NR	NR	9.6	NR	NR	NR	3.8	NR	Negative	NR	Yes (3.4 IU/g Hb)
Hasler J and Lee S [38]	46	M	Raw fava beans	1	NR	NR	NR	Middle Eastern	Dark urine Jaundice Malaise Nausea Vomit	BT: 100.6 °F BP: 120/70 mmHg HR: 104 bpm RR: 18/min	5.4	NR	15.8	21	Total: 7.5	NR	NR	Yes (whole blood)	NR
Hampl JS et al. [39]	34	M	Fava beans	1	NR	NR	NR	Iraqi	Anemia Dark urine Jaundice Renal failure Vomit	BT: 99.3 °F BP: 151/67 mmHg HR: 117 bmp RR: 18/min	3.6	900,000	9	NR	2.98	NR	N	Yes (packed RBCs)	Yes (2.6 U/g Hb)
Lim F et al. [40]	44	F	Boiled fava beans	1–2	NR	NR	No	NR	Dark urine Edema Malaise Jaundice	BP: 110/90 mmHg HR: 100 bmp	5.5	NR	NR	133 × 10^9^/L	2.81	NR	NR	Yes (whole blood)	Yes (2.5 U/g Hb)
Soyuncu S et al. [41]	56	M	Fava beans	3	NR	No	Don’t know	NR	Dizziness Syncope	BT: 37.2 °C BP: 130/80 mmHg HR: 116 bmp RR: 20/min	8.4	NR	23.7	2.8	Total:8.53 Indirect: 0.39	NR	Negative	Yes (packed RBCs)	Yes
Ata F et al. [42]	56	M	Fava beans	NR	Yes	NR	NR	Qatari	Dizziness Dyspnea Jaundice Pallor Vomit	BP: 136/76 mmHg HR: 93 bpm Sat02: 70%	9.9	NR	NR	NR	Indirect: 3.51	NR	NR	Yes (packed RBCs)	Yes (23 mU/10^9^ RBC)
Al-Dubai H et al. [43]	47	M	Fava beans	2	NR	NR	NR	NR	Dark urine Jaundice	BT: 38.5 °C BP: 125/78 mmHg HR: 117 bpm RR: 20/min Sat02: 88%	12	NR	NR	17.8	Total: 4.89 Direct: 0.65	NR	NR	No	Yes (24 mU/10^9^ RBC)
Hartigan JD and Gurnett TJ [44]	74	M	Fava beans	0–1	Yes, twice	No	NR	NR	Dark urine Hepatomegaly Jaundice Malaise	BT: 98.6 °F BP: 120/60 mmHg HR: 80 bpm	8.3	NR	25	NR	NR	NR	Negative	Yes (whole blood)	NR
Torres CD et al. [45]	67	M	Beans	NR	NR	NR	No	NR	Abdominal pain Jaundice Malaise Renal failure	NR	4.9	NR	14.1	4.6	Total: 5.7 Indirect: 3.2	NR	Negative	Yes (packed RBCs)	Yes

Legenda: BP, blood pressure; BT, body temperature; Hb, hemoglobin; HR, heart rate; Ht, hematocrit; NR, not reported; RBC, red blood cell; RR, respiratory rate; Sat02, saturation on room air. Clinical parameters were evaluated according to the specific laboratory ranges given in the cited article.

## Data Availability

Not applicable.
